# 加速康复外科和日间手术模式在胸外科中的应用现状及发展前景

**DOI:** 10.3779/j.issn.1009-3419.2020.102.08

**Published:** 2020-09-20

**Authors:** 诚 沈, 帅 常, 坤 周, 国卫 车

**Affiliations:** 610041 成都，四川大学华西医院胸外科 Department of Thoracic Surgery, West China Hospital, Sichuan University, Chengdu 610041, China

**Keywords:** 加速康复外科, 日间手术, 胸外科, 围术期, Enhanced recovery after surgery, Day surgery, Thoracic surgery, Perioperative period

## Abstract

加速康复外科理念联合微创外科技术的临床实施所取得的良好效果体现在降低围手术期并发症的发生率并缩短住院时间，已广泛应用于不同专业的外科领域。日间手术模式的实施可缩短患者等待手术时间，减轻经济负担，对于胸外科而言，在医院等待的时间越短，越有利于患者身体以及心理恢复。随着微创技术和加速康复流程的广泛实施，加速康复外科在胸外科手术中的应用使部分胸外科手术在日间病房完成成为可能，同时日间胸外科手术也是加速康复外科理念实施的集中体现。本文就加速康复外科在胸外科手术领域的应用现状和日间手术模式在我国的发展前景做一综述。

## Abstract (en)

The good results of enhanced recovery after surgery (ERAS) combined with minimally invasive surgery are reflected in reducing the incidence of perioperative complications and shortening the length of hospitalization (LOS). It has been widely used in surgical fields of different specialties. The implementation of the day surgery mode can shorten the waiting time for patients and reduce the financial burden. Especially in thoracic surgery, the shorter the waiting time in the hospital, the more beneficial for the patient's physical and psychological recovery. With the widespread implementation of minimally invasive technology and accelerated rehabilitation procedures, the application of ERAS in thoracic surgery has made it possible for some thoracic surgery to be completed in the day surgery. This article summarizes the current application of ERAS in the field of thoracic surgery and the development prospects of day surgery models in China.

加速康复外科（enhanced recovery after surgery, ERAS）又称为快速康复外科（fast-track surgery, FTS），最早是1997年由丹麦学者Kehlet提出的^[1]^。其内涵是减少创伤对机体应激反应，促进机能快速康复；外延体现在临床上降低并发症发生率并缩短住院时间^[2, 3]^。近年来随着ERAS发展迅速，让部分患者实行日间手术成为可能。本文就ERAS在胸外科手术领域的应用现状和日间手术在我国的发展前景做一综述。

## 1 ERAS与胸外科

在早期，ERAS理念更多体现在普通外科手术患者围术期诊治流程中的优化^[4]^。随后，基于腹腔镜微创技术的应用，其在ERAS流程中的积极作用突显，微创手术自身相较于传统手术方式所具有的创伤小、疼痛轻、患者术后恢复快及住院时间短的特点，不仅降低了外科手术对患者造成的应激反应和并发症，也明显提高了术后患者的满意度^[5]^。因此ERAS理念早期多应用于骨科、泌尿外科和妇科等科室。从2006年至今，ERAS理念在胸外科的临床应用也逐步得到开展，尤其是在胸外科患者围术期的管理路径优化方面得到了更为普遍的革新，理念所倡导的多模式治疗方法和多学科协作已得到认可^[6-8]^，如2016年中国加速康复外科专家组推出的《中国加速康复外科围手术期管理专家共识（2016版）》及随后的《加速康复外科中国专家共识及路径管理指南（2018版）》和胸外科领域推出的《多学科围手术期气道管理专家共识（2018年版）》^[9-11]^。

普外科围术期常规的ERAS措施同样可以应用于胸外科患者，比如：术前宣教、饮食管理、麻醉的评估和优化、术中控液、管道的管理、多模式镇痛方案和术后常见并发症的预防等^[12-14]^。其次，针对胸外科患者围术期相关的特殊准备需要采取相应的措施，包括：①术前呼吸道准备：对于吸烟患者，建议至少完全戒烟2周以上，最好是4周；对于有800年支以上吸烟史的中重度慢性阻塞性肺病患者，建议术前进行药物联合物理康复的综合肺康复训练^[15, 16]^。具体包括了消炎、平喘，雾化吸入糖皮质激素类或支气管扩张剂等药物治疗；激励式肺量计吸气训练、静态肺功能检测（pulmonary function test, PFT）结合爬楼试验，心肺运动试验或6 min步行试验等物理锻炼来评估患者肺功能情况^[17, 18]^。②管道的管理：一方面是关于胸外科患者围术期导尿管的管理。胸外科围术期留置尿管的原因主要是术中观察尿量，以便于控制术中液体过多或过少；术后预防因麻醉导致尿潴留。然而，留置尿管不但会引起患者不适，也易导致术后清醒时患者麻醉期苏醒期躁动和不良事件，术后尿路感染和降低患者舒适度，并限制术后早期活动。有研究^[19-21]^表明，手术时间缩短、失血量减少、术中输液量的合理应用及麻醉药的个体化应用、使术中患者的尿量约在正常膀胱容量之内，故部分符合筛选标准的患者术中可以不留置尿管。多项研究^[22, 23]^结果提示，肺癌肺叶切除患者术后发生尿潴留的概率约为10%。尽管在临床实际运用中，术前宣教和术后指导及诱导排尿从护理工作量的角度看可能会有所增加，但由于不再安置尿管，同时节省了留置尿管后的日常护理工作，大大降低了护士在管道护理方面的工作负荷^[24, 25]^。

另一方面是关于胸外科患者围术期胸腔引流管的管理。传统的胸腔引流方法是两根引流管，该方法主要强调了充分排出胸腔积气积液同时促使肺复张，但其缺点也不可无视，既不利于患者排痰，也不利于患者下床活动和术后物理康复训练等^[26, 27]^。随着单胸腔引流管引流在胸腔镜肺叶切除术（video-assisted thoracic surgery, VATS）中的实施，大部分临床试验结果均取得了与双管引流同样的效果，且单引流管在术后舒适度和快速康复中显示出明显优势^[21, 26]^。早期的单胸腔引流管以28 F口径的硅胶管为主，虽然有助于术后肺复张，但其自身仍存在很多问题，例如引流管口周围容易渗液或漏气，管径粗不易密闭；术后需要留预置线，以利拔管后封闭引流管口；预置线结扎后易导致皮肤坏死、感染，延迟切口愈合；引流管口常常以瘢痕方式愈合，需要长期换药而给患者带来不适^[28, 29]^。有研究^[26]^报道，可选用16 F尿管代替28 F引流管进行引流。其优势主要体现在：①没有增加术后与引流管密切相关的并发症（如住院期间和30 d后胸腔积液或积气）；②术后引流量显著减少；③改善了术后第3天的肺功能；④引流持续时间、管口拆线时间及术后住院时间显著缩短；⑤引流管口Ⅰ级愈合率接近100%。基于最新的研究进展，无管化（Tubeless）理念的提出，更是把ERAS的围术期管理模式在胸外科患者住院期间得以优化和改善^[30, 31]^。ERAS作为一种新的理念，是对传统临床实践经验的系统性改变，但实施的过程中仍需要多学科的支持和质量控制^[32, 33]^。

## 2 日间手术模式与胸外科

日间手术（day surgery或ambulatory surgery）最早是在1909年由James Nicoll报道的^[34]^。随后，日间手术在全球尤其是欧美发达国家的日间手术量已达到其医院手术总量的80%以上。国际日间手术协会（International Association for Ambulatory Surgery, IAAS）推荐的日间手术定义是：“患者在同一个工作日完成手术或操作并出院的，不包括那些在诊所或门诊进行的手术或操作”^[35]^。2015年10月15日，由国家卫生计生委卫生发展研究中心支持和指导发起成立的中国日间手术合作联盟（China Ambulatory Surgery Alliance, CASA）正式推出中国日间手术定义：“日间手术指患者在一日（24 h）内入、出院完成的手术或操作。”

ERAS理念在胸外科手术中的成功实施，使部分胸外科手术在日间病房完成成为可能，同时胸外科手术日间化也是ERAS理念实施的更进一步的集中体现。微创外科技术和围术期加速康复流程的广泛实施提高了优质医疗资源的使用效率^[36]^。相对于住院手术，ERAS理念下的胸外科日间手术具有明显的优势：不仅减少院内感染，尤其是耐药菌感染的机会，而且术后回到相对舒适的家庭环境更有利于患者自身术后的康复。此外，从卫生经济学角度出发，日间手术还明显降低了患者的整体住院费用，也实现了政府、保险公司和患者多赢，同时也节省了有限的医疗资源，让更多需要手术符合手术指征的患者有机会进行治疗^[37-39]^。

目前有关胸外科手术的临床研究主要是针对肺部良性肿瘤和纵隔肿瘤切除、手汗症的治疗及原发性气胸的手术处理。赵伟军等^[40]^回顾性分析了31例良性纵隔神经源性肿瘤患者，日间手术组采用保留自主呼吸麻醉的胸腔镜手术方式，通过比较日间手术组和常规手术组间手术时间、术中出血量、术中血氧饱和度、麻醉复苏时间、术后引流量、术后住院时间等指标发现，日间手术的患者在麻醉准备时间、复苏时间、术后引流量、住院时间指标方面均优于常规手术组（均*P* < 0.05），这样的临床结果充分体现了日间手术联合ERAS理念在胸外科良性纵隔肿瘤切除术中实施的意义。其次，日间手术也应用于手汗症患者的临床处理。研究^[41]^提示，回顾性分析52例在日间手术模式下行双侧交感神经切断术的患者与常规手术模式下，在术后疼痛评估和住院费用方面的比较。日间手术组的优势明显体现在降低了术后疼痛的症状，患者满意度明显得到提升。同样的，日间手术模式在原发性气胸患者中也得到了推广和应用^[42]^。

国外的临床研究者和医疗机构也在逐步探索和寻找胸外科手术在日间模式下的方向。最早有关肺叶切除手术实施日间模式的是来自Tovar等的临床研究^[43]^，10例接受小切口辅助开胸肺叶切除手术患者中，有2例患者术后当天晚上经评估后即拔出胸引管，6例患者术后第一天早上拔出胸引管。10例患者术后均未出现相关并发症。随后，在2001年Tovar团队^[44]^在其前期研究的基础上70岁年龄作为分界点，分别比较了 < 70岁（年轻组）和≥70岁（老年组）患者在实施胸腔镜辅助小切口肺叶切除术中，两类患者在实施日间模式的可行性及整体评估患者的平均住院时间和术后并发症的发生率及死亡率。研究共纳入65例患者，其中≥70岁患者共30例。所有患者中仅3例患者出现术后并发症。其中老年组有1例术后出现肺气肿，而年轻组中1例患者表现为术后声带麻痹及1例出现长期漏气而需要延长胸引管带管时间。研究结果充分说明了微创外科技术和精准切除以及围术期流程管理优化的现代外科理念为ERAS在胸外科日间手术开展和实施奠定了理论与实践基础，同时也确保不同年龄段人群出院后的安全和康复，在有效节约资源的情况下，全方位保证患者质量安全。法国和英国的研究者也报道了有关其首次开展胸外科日间手术的临床研究。手术均以全胸腔镜操作为基础，包括了肺部良恶性结节的切除、肺活检术及胸壁手术^[34, 45]^。

尽管胸外科日间手术模式已经逐步开展起来，但患者的安全性依然是最重要也是必须考虑的问题^[46]^。肺术后出血和肺持续漏气是重要且常见的并发症。虽然行胸外科日间手术患者大多数于手术当天下午或晚上拔出胸引管，但密切观察引流量及严格评估拔管指针依然是及时发现肺术后出血的重要方法。针对肺部持续漏气的处理，术前应加强对患者、家属及其护理者进行全面的教育和依从性评估，同时，选择合适的胸外科患者进行日间手术^[47, 48]^。患者的选择包括疾病因素和患者自身条件的评估，应由外科医师、麻醉医师、专科护士和看护者等共同完成。患者术前的全身情况，其他合并症（高血压、糖尿病等）均会对术后的恢复造成影响。全面评估患者的生理、精神状态和生活自理能力也非常重要。术中应对余肺断面严密闭合或给予防漏气生物材料。术后可给予低压力负压吸引。

## 3 前景和展望

随着ERAS理念逐步深入和应用到胸外科领域，必将对胸外科的围手术期处理产生更大影响。ERAS的理念注重患者的治疗效果，而不仅仅是速度。围术期处理措施的施行必须在循证医学或真实世界数据或证据指导下进行，以使患者受益为目的。ERAS方案的重点在于经过合理的处理措施，患者并发症发生率降低，在此基础上术后住院时间才能安全缩短^[49, 50]^。

日间手术在国外发达国家发展如火如荼，纳入日间手术的种类已普遍较多，有较完善的诊疗护理和康复体系。我国的日间诊疗模式开展成效初显，实践证明按临床路径进行规范化管理，融入ERAS理念的胸外科日间手术模式是一种安全、有效的手术模式。同时，我们可以借鉴国外相关的管理经验，制定符合我们自身胸外科日间手术的纳入排除标准及各种规章制度，提高技术及管理水平，完善相关保障体系，保证患者的医疗护理安全和质量，进一步促进胸外科日间手术模式的健康发展。
